# Spectrum analysis of digital UPWM signals generated from random modulating signals

**DOI:** 10.1038/s41598-024-54983-0

**Published:** 2024-02-22

**Authors:** Konstantinos Kaleris, Emmanouil Psarakis, John Mourjopoulos

**Affiliations:** 1 Audio and Acoustic Technology Group, Wire Communications Laboratory, Department of Electrical & Computer Engineering, University of Patras, Rion Campus, 26500 Patras, Greece; 2 Institute of Plasma Physics and Lasers, Hellenic Mediterranean University, Tria Monastiria, 74100 Rethymno, Greece; 3 Department of Computer Engineering & Informatics, University of Patras, Rion Campus, 26500 Patras, Greece

**Keywords:** Statistics, Electrical and electronic engineering

## Abstract

This work studies the spectrum of discrete-time Uniform-sampling pulse width modulation (UPWM) signals originating from stochastic input signals. It demonstrates that for ergodic input sequences of independent and identically distributed random variables, the Discrete Fourier Transform (DFT) of the UPWM signals can be directly estimated from the input signal’s statistics. Consequently, it is shown that if the input signal can be modeled as such a random sequence, only statistical information of the sequence is required for the accurate estimation of the DFT of the UPWM signal. This is achieved here by proving that the DFT estimators obtained by observation of the input sequence within a time window are consistent estimators of the DFT coefficients of the underlying random process. Moreover, for signals whose generalized probability density functions can be expressed as functions of a small number of parameters, the DFT coefficients can be estimated or even calculated via closed-form expressions with linear complexity. Examples are given for input signals derived from symmetric and asymmetric distributions. The results are validated by comparison with evaluations of the UPWM signal’s DFT via the Fast Fourier Transform (FFT). The proposed method provides a mathematical framework for the analysis and design of UPWM systems whose inputs have known statistical properties.

## Introduction

Pulse Width Modulation (PWM) encodes signal information in the duty cycle of rectangular pulses. Hence, PWM signals constitute sequences of rectangular pulses with fixed amplitude and a duty cycle that depends on the instant amplitude of the modulating signal. PWM is widely adopted in telecommunications, signal processing, audio technology and power systems, among others, as it provides significant benefits over traditional analog implementations. In telecommunications it is used for efficient signal coding and transmission, i.e., in burst-mode RF transmitters^1–3^ and Power Line Communication (PLC) systems^4^. In audio technology, it is used in class-D amplifiers for high-efficiency signal amplification^5–7^ and for efficient digital-to-analog conversion of audio streams^8^. It is also adopted in a wide range of electrical engineering applications such as power electronics^9,10^, Wireless Power Transmission (WPT) systems^11,12^, electric motor control^13,14^ and others. Uniform-sampling PWM (UPWM) is mainly used in digital PWM systems and computational analysis and processing of PWM signals. In UPWM, a discrete-time and quantized modulating signal with k-bit resolution results to PWM pulses with a k-bit quantized width, accounting for the different signal amplitudes. Due to its digital nature, UPWM is utilized in computational simulations while, under conditions, it can be used to approximate analog Natural-Sampling PWM (NPWM) signals^15^ with satisfactory accuracy^6,15–17^. Specifically, the UPWM signal tends to the respective NPWM signal with (a) decreasing UWPM quantization step and signal amplitude and (b) with increasing UPWM oversampling factor, namely the use of a UPWM carrier frequency that is a multiple of the minimum required carrier frequency, as presented in “Deterministic input sequences” section.

PWM systems generally suffer from out-of-band modulation products, with well-known hazards that mainly lie in the damage of components due to overheating, non-linear behavior, noise through electromagnetic interference (EMI) etc.^18–20^. For example, in PWM-based switch mode power supplies and class-D amplifiers, power signal delivery is usually done through a low-pass filter that removes the excessive high-frequency energy^18,21^ at the expense of increased cost, weight and dimensions of the system^22^. Filterless implementations i.e., of Class-D amplifiers have also been proposed, in which the amplified PWM signal is directly delivered on the load, namely the loudspeaker^22,23^. For the design and implementation of such PWM systems, the precise estimation of the PWM frequency spectra, and especially the out-of-band modulation products, is critical.

The estimation or calculation of the PWM frequency spectra from the input signal has been a matter of research since decades and several solutions have been proposed. One of the pioneering works published in 1933 by Bennet^24^, described modulation products via the double Fourier Series method. Bennet’s method is limited to modulating signals including a small number of frequencies, which is nevertheless useful in applications such as power electronics. In 2003, Song et al.^15^ and later Deslauriers et al., Deng et al., Kostic et al. and Tanovic et al.^25–28^, presented general methods for the calculation of modulation products of PWM signals generated by arbitrary analog modulating signals, in the form of infinite sums. The frequency spectra of digital PWM schemes have also been studied in various works i.e., by Floros et al.^6^ and Vogel et al.^2,29^. In another approach, PWM-coded signals have been probabilistically modeled in the time-domain and evaluated in terms of stationarity, autocorrelation and power spectral density^30^. Estimation of the PWM power spectrum using classical spectral estimation techniques, such as the Bartlett and Welch methods, has been proposed for the case of Random PWM (RPWM) signals^31,32^, namely sequences of PWM pulses with dither. Moreover, in the past 20 years it has been shown that various types of UPWM input signals can be modeled via their statistical characteristics as sequences of independent and identically distributed (i.i.d.) random variables (RVs). Apart from the sinusoidal signals that follow the arcsine distribution, speech signals modeled as sequences of truncated Laplacian RVs^33–35^, music signals^36^, sonar signals^37^ and of course, noise signals modeled via the Gaussian or truncated Gaussian distribution, are common examples where statistical signal modeling has led to significant benefits and advancements in the analysis and digital signal processing.

In this work, and under the assumption of statistically modeled input signals, we focus on the relation between the input signal statistics and the frequency spectrum of the resulting digital UPWM signal. Ultimately, we develop a novel mathematical framework for the description of UPWM systems solely based on statistical information of the input signals. For this purpose, input or modulating signal refers to a sequence of i.i.d RVs, UPWM spectra refers to the RVs obtained via discrete Fourier transform (DFT) of the UPWM signal and statistical characteristics of the modulating signal refers to the mean, variance and higher order moments of the underlying distribution of the input sequence, also mentioned as amplitude distribution or, simply, distribution. Here, it is shown for the first time that the expected values of the DFT coefficients of a UPWM signal can be precisely estimated from the statistics of the input sequence, having a zero value on the out-of-band side frequencies and a generally non-zero value on the carrier harmonic frequencies. The analysis reveals that odd carrier harmonics directly relate to even input distribution moments and even harmonics relate to odd input distribution moments. Importantly, for input signal distributions whose moments can be expressed in terms of a few parameters e.g., the variance, closed-form formulas are derived for the estimation of the true values of the DFT coefficients, a fact that significantly facilitates and accelerates computations of PWM frequency spectra. The findings are validated by comparison with computational evaluations of the DFT coefficients for various UPWM test signals. Finally, application of the presented DFT analysis on a real speech signal modeled as a sequence of i.i.d. RV is examined. The very good agreement of the proposed analytical method with the computational evaluations highlights its applicability in the efficient design of real-world PWM systems.

## Discrete-time UPWM

In this section, Uniform-sampling Pulse Width Modulation of digital signals and their discrete Fourier transform are described. The classical UPWM conversion of deterministic Pulse Code Modulation (PCM) input signals is extended towards UPWM conversion of random sequencies and the DFT spectra of the resulting random UPWM signals are derived.

### Deterministic input sequences

**Figure 1:**
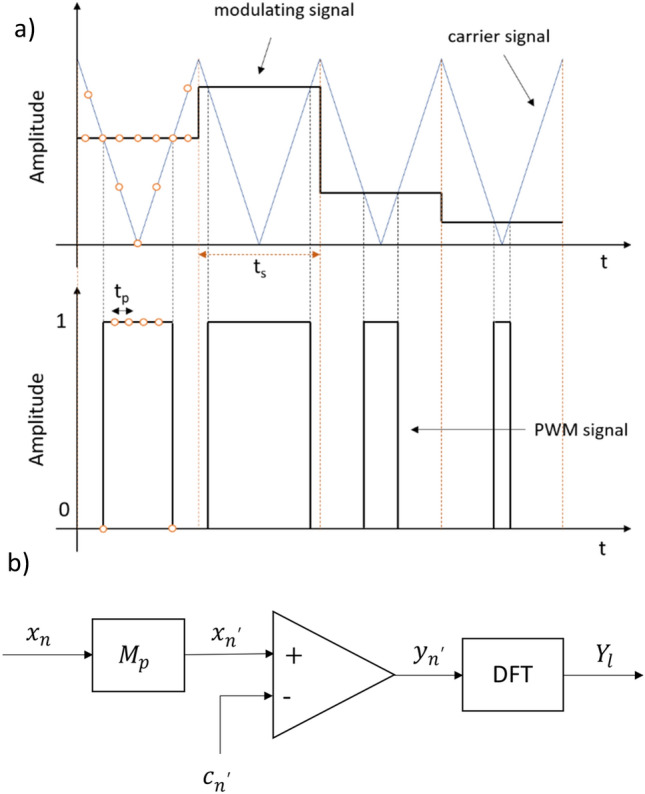
(**a**) Signal representation and (**b**) block diagram of the conversion of a digital modulating signal to a stream of discrete-time UPWM pulses by comparison to a triangular carrier.

Let a discrete-time signal ${x}_{n}:{x}_{n}\in \left[-\frac{1}{2} ,\frac{1}{2}\right],n\in \left[0,d-1\right]$, quantised with *k*-bit resolution, sampling frequency ${f}_{s}$, sampling period ${t}_{s}$ and duration ${t}_{d}=d{t}_{s}$, be the input to a UPWM conversion process (For simplicity, the signal is assumed to have an even number of samples $d.$). The resulting discrete-time, double-edge UPWM signal ${y}_{{n}^{\prime}}:{y}_{{n}^{\prime}}\in \left[\text{0,1}\right], {n}^{\prime}\in \left[0,D-1\right]$, is a stream of square pulses of equal amplitude and varying width. A schematic representation of the conversion process from ${x}_{n}$ to ${y}_{{n}^{\prime}}$ can be seen in Fig. 1a. Each UPWM pulse corresponds to a single sample of ${x}_{n}$ and is centered within the sampling interval ${t}_{s}$. The pulses take ${2}^{k}$ possible discrete widths, corresponding to the discrete amplitudes of ${x}_{n}$. Each pulse is represented by ${M}_{p}=2({2}^{k}-1)$ samples^13^, with a sampling period ${t}_{p}=\frac{{t}_{s}}{{M}_{p}}$, or equivalently a sampling frequency ${f}_{p}={M}_{p}{f}_{s}$. The total number of samples of the UPWM signal is $D={M}_{p}d=\frac{{t}_{d}}{{t}_{p}}$ while its duration remains the same as the input, that is ${t}_{d}$. The UPWM pulses result from comparison of the instant amplitude of the upsampled—to the frequency ${f}_{p}$—modulating signal ${x}_{{n}^{\prime}}$ with the amplitude of a triangular carrier ${c}_{{n}^{\prime}}$ with fundamental frequency ${f}_{{\text{car}}}={f}_{s}$ as shown in the block diagram of Fig. 1b.

**Figure 2:**
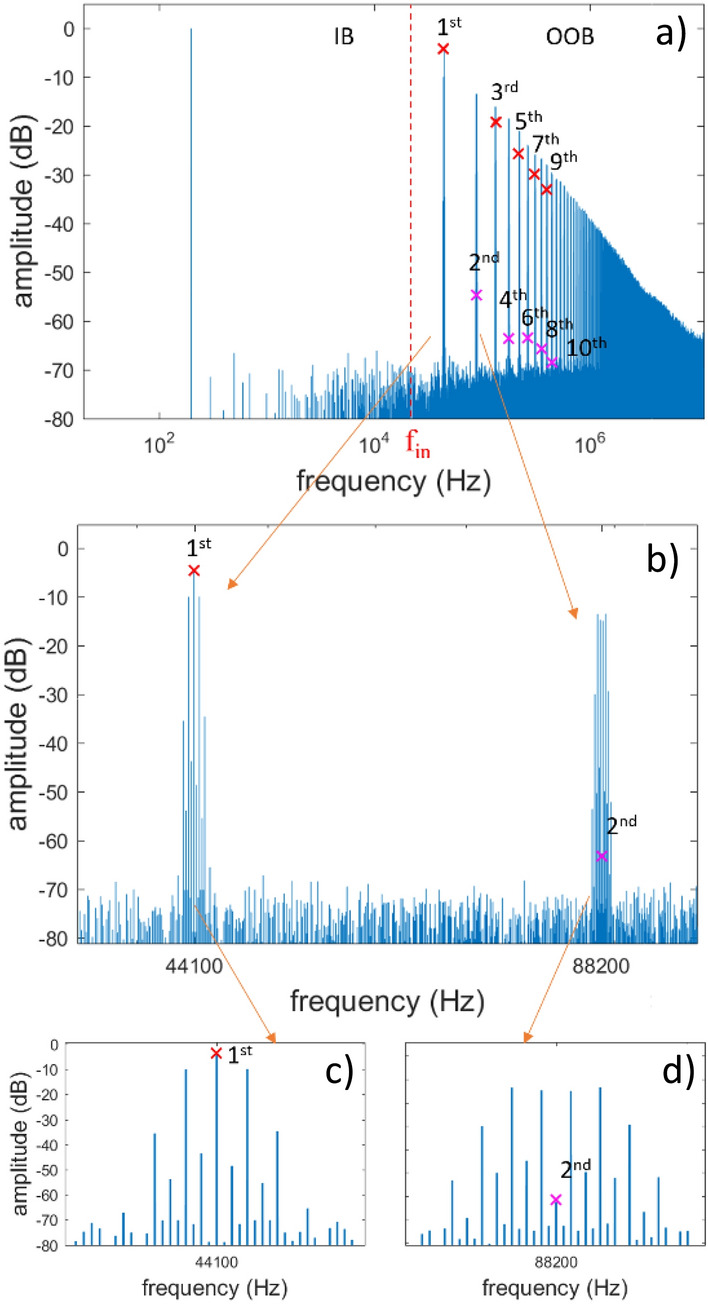
Computationally evaluated DFT of a sinewave modulating signal with ${f}_{{\text{sin}}}=200 \, {\text{Hz}}$; ${f}_{s} = 44.1 \, {\text{kHz}}$ and 8-bit resolution: (**a**) full DFT spectrum, (**b**) detail from 1st and 2nd carrier harmonic, (**c**) carrier fundamental and surrounding side frequencies and (**d**) carrier 2nd harmonic and surrounding side frequencies. Odd/Even harmonics marked with crosses.

In the discrete frequency domain, ${x}_{n}$ and ${y}_{{n}^{\prime}}$ are described by the complex DFT vectors ${X}_{k}:k\in \left[0,\frac{d}{2}\right]$ and ${Y}_{l}:l\in \left[0,\frac{D}{2}\right]$ respectively; for the purpose of the presented analysis, the negative frequencies will be neglected. The vectors ${X}_{k}$ and ${Y}_{l}$ have a frequency resolution ${f}_{d}=\frac{1}{{t}_{d}}$ and Nyquist frequencies $\frac{{f}_{s}}{2}$ and $\frac{{f}_{p}}{2}$ respectively. The frequency range up to $\frac{{f}_{s}}{2}$ is mentioned as the band of interest, or in-band range, whereas the range $\frac{{f}_{s}}{2}\le f<\frac{{f}_{p}}{2}$ is mentioned as out-of-band range. The out-of-band components of ${Y}_{l}$ are here treated distinctly for the carrier frequencies, namely the carrier fundamental frequency and its harmonics ${f}_{m}=m{f}_{s}$, $m\in {\mathbb{N}}:m\in \left[1, \frac{{M}_{p}}{2}-1\right]$ and the side frequencies ${f}_{m,b}$, with $b\in \left[-\frac{d}{2}+1,\frac{d}{2}\right]\backslash \{0\}$ defined as the out-of-band frequencies around the carrier harmonics. Out-of-band frequencies are addressed via the nearest carrier harmonic ${f}_{m}$ and a frequency distance ${f}_{b}=b{f}_{d}=\frac{b}{d}{f}_{s}$ from ${f}_{m}$, so that ${f}_{m,b}={f}_{m}+{f}_{b}$; the related DFT index is ${l}_{m,b}=md+b$. Figure 2 illustrates the DFT representation of a UPWM modulated digital sinewave ${x}_{n}=\frac{1}{2}{\text{sin}}(2\pi {f}_{{\text{sin}}}n{t}_{s})$ with ${f}_{{\text{sin}}}=200$ Hz and sampling frequency ${f}_{s}=44.1$ kHz. In Fig. 2a, the in-band and out-of-band components are separated by the frequency $\frac{{f}_{s}}{2}$ and the carrier components are marked with crosses. Figure 2b–d present details from the spectra around the carrier fundamental ${f}_{1}=44.1$ kHz (1st harmonic) and 2nd harmonic ${f}_{2}=88.2$ kHz.

Now, the DFT coefficients ${Y}_{l}$ of the UPWM signal ${y}_{{n}^{\prime}}$ at a frequency $f=l{f}_{d}$ are commonly expressed in terms of the samples of the UPWM signal as:

1 $${Y}_{l}=\frac{1}{D}\sum \limits_{{n}^{{{\prime}}}=0}^{D-1}{y}_{{n}^{{{\prime}}}}{e}^{-j\frac{2\pi l}{D}{n}^{{{\prime}}}}.$$

Floros et al.^6^ have shown that ${Y}_{l}$ can be directly calculated from the samples of the input signal ${x}_{n}$, according to:

2 $${Y}_{l}={A}_{l}\sum \limits_{n=0}^{d-1}{\text{sin}}\left(\frac{\pi l}{d}\left({x}_{n}+\frac{1}{2}\right)\right){e}^{-j\frac{2\pi l}{d}n },$$

where ${A}_{l}=\frac{1}{\pi l}{e}^{-j\frac{\pi l}{d}}$. Equation (2) requires the knowledge of the input time-series for the calculation of the coefficients ${Y}_{l}$. In this work it is shown that when the input signal ${x}_{n}$ is an ergodic random sequence of i.i.d RV, only statistical information of the amplitude distribution of ${x}_{n}$ is required for the precise estimation of the coefficients ${Y}_{l}$.

### Random input sequences

By adopting the vector notation for the random input sequences, let ${{\varvec{x}}}_{d}\left(\theta \right)=[{x}_{0}\left(\theta \right) {x}_{1}\left(\theta \right)\dots {x}_{d-1}(\theta ){]}^{t}$ be a random vector containing $d$ i.i.d. RV ${x}_{n}(\theta )$ with $n\in [0,d-1]$ taking values within the range ${x}_{n}(\theta )\in \left[-\frac{1}{2} ,\frac{1}{2}\right]$ and quantised with *k*-bit resolution. Let us also consider that the functions ${f}_{{x}_{n}}\left({\text{x}}\right)$ and ${{\text{F}}}_{{x}_{n}}\left({\text{x}}\right)$ denote the Generalized Probability Density Functions (GPDF) and the Cumulative Distribution Function of ${x}_{n}(\theta )$ respectively. Then, it is well-known that the average of the elements of ${{\varvec{x}}}_{d}(\theta )$:

3 $${w}_{d}\left(\theta \right)=\frac{1}{d}<{1}_{d},{{\varvec{x}}}_{d}\left(\theta \right)>,$$

with <.,.> and ${1}_{d}$ denoting the inner product operator and an all-ones vector of length $d$ respectively, defines a new RV ${w}_{d}(\theta )$ whose GPDF ${{\text{f}}}_{{w}_{d}}\left({\text{w}}\right)$ is given by:

4 $${f}_{{w}_{d}}\left({\text{w}}\right)={f}_{{x}_{1}}\left({\text{x}}\right)\times {f}_{{x}_{2}}\left({\text{x}}\right)\times \cdots \times {f}_{{x}_{d}}\left({\text{x}}\right),$$

where ‘*’ denotes the linear convolution operator. In addition, the following relations hold for ${w}_{d}\left(\theta \right)$:

5 $${\mu }_{{w}_{d}}={\mathbb{E}}\left[{w}_{d}\left(\theta \right)\right]={\mu }_{x},$$

and

6 $${\sigma }_{{w}_{d}}^{2}={\mathbb{E}}[({w}_{d}\left(\theta \right)-{\mu }_{x}{)}^{2}]=\frac{{\sigma }_{x}^{2}}{d},$$

where ${\mathbb{E}}[.]$, ${\mu }_{x}$, ${\mu }_{{w}_{d}}$, ${\sigma }_{x}^{2}, {\sigma }_{{w}_{d}}^{2}$ denote the expectation operator and the means and variances of the RV ${x}_{n}\left(\theta \right), {w}_{d}(\theta )$ respectively. Note that from Eq. (6) that, as the length $d$ of the random vector ${{\varvec{x}}}_{d}\left(\theta \right)$ tends to infinity the variance of the RV ${w}_{d}\left(\theta \right)$ tends to zero, i.e:

7 $$\underset{d\to \infty }{{\text{lim}}}{\sigma }_{{w}_{d}}^{2}=\underset{d\to \infty }{{\text{lim}}}{\mathbb{E}}[({w}_{d}\left(\theta \right)-{\mu }_{x}{)}^{2}]=0.$$

For Eq. (7), the law of large numbers ensures that ${w}_{d}\left(\theta \right)$ is a consistent estimator of the expected value of the i.i.d. random variables ${x}_{n}\left(\theta \right), n=\text{1,2},\dots ,d$.

Let us consider now that the random vector ${{\varvec{x}}}_{d}\left(\theta \right)$ is an input in a UPWM conversion process. According to Eq. (2), the Discrete Fourier Transform coefficients ${Y}_{l}\left(\theta \right)$ of the UPWM vector ${y}_{n^{\prime}}$ are given by:

8 $${Y}_{l}\left(\theta \right)={e}^{-j\frac{\pi l}{d}}\frac{1}{\pi l}\sum_{n=0}^{d-1}{\text{sin}}\left(\frac{\pi l}{d}\left({x}_{n}\left(\theta \right)+\frac{1}{2}\right)\right){e}^{-j\frac{2\pi l}{d}n }.$$

The RV ${Y}_{l}\left(\theta \right)$ can be expressed using Eq. (3) as:

9 $${Y}_{l}\left(\theta \right)={e}^{-j\frac{\pi l}{d}}\frac{1}{\pi l}<{{\varvec{e}}}_{d,l},{{\varvec{v}}}_{d,l}\left(\theta \right)>,$$

where ${{\varvec{e}}}_{d,l}={\left[1 {e}^{-j\frac{2\pi l}{d} } \dots {e}^{-j\frac{2\pi l}{d} \left(d-1\right)}\right]}^{t}$ and ${{\varvec{v}}}_{d,l}\left(\theta \right)$ is a vector of i.i.d. RVs ${v}_{n,l}$ resulting from the transformation of the RVs ${{\varvec{x}}}_{d}\left(\theta \right)$, i.e.:

10 $${{\varvec{v}}}_{d,l}\left(\theta \right)=[{v}_{0,l}\left(\theta \right) {v}_{1,l}\left(\theta \right)\dots {v}_{d-1,l}\left(\theta \right){]}^{t}={\text{g}}\left({a}_{l}{{\varvec{x}}}_{d}\left(\theta \right)+{b}_{l}\right),$$

with ${\text{g}}\left(\cdot \right)$ being the sinusoidal function acting on each element of ${{\varvec{x}}}_{d}\left(\theta \right)$ and ${a}_{l}=\frac{\pi l}{d}$, ${b}_{l}=\frac{ {a}_{l}}{2}$.

In the next paragraph, it is demonstrated that for random input sequences of i.i.d. RVs, the variables ${Y}_{l}\left(\theta \right)$ defined in Eq. (9) constitute consistent estimators of the expected value. Carrier harmonics and the side frequencies are treated separately.

#### Carrier harmonics

For the carrier harmonics, ${e}^{-j\frac{2\pi l}{d}n }={e}^{-j2\pi mn}=1$, and Eq. (8) reduces to:

11 $${Y}_{md}\left(\theta \right)=\frac{{A}_{m}}{d}<{1}_{{\varvec{d}}},{{\varvec{v}}}_{d,md}\left(\theta \right)>,$$

with ${A}_{m}=\frac{{\left(-1\right)}^{m}}{\pi m}$. The expected value ${\mu }_{{Y}_{md}}$ and the variance ${\sigma }_{{Y}_{md}}^{2}$ of the variables ${Y}_{md}(\theta )$ are respectively given by:

12 $${\mu }_{{Y}_{md}}=\frac{{A}_{m}}{d}{\mathbb{E}}\left[<1,{{\varvec{v}}}_{d,md}\left(\theta \right)>\right]={A}_{m}{\mu }_{{v}_{md}},$$

and

$${\sigma }_{{Y}_{md}}^{2}=\frac{1}{{\left(\pi m\right)}^{2}}{\mathbb{E}}[\left(\frac{1}{d}<1,{{\varvec{v}}}_{d,md}\left(\theta \right)>-{\mu }_{{v}_{md}}{)}^{2}\right],$$

which by using Εqs. (3) and (6) becomes:

13 $${\sigma }_{{Y}_{md}}^{2}=\frac{{\sigma }_{{v}_{md}}^{2}}{{{\pi }^{2}m}^{2}d}.$$

Since ${\sigma }_{{v}_{md}}^{2}$ is bounded and $\underset{d\to \infty }{{\text{lim}}}{\sigma }_{{Y}_{md}}^{2}=0$, the coefficients ${Y}_{md}\left(\theta \right)$ constitute consistent estimators of the true mean.

#### Side frequencies

Let us now concentrate on the side frequencies where the related DFT index takes the values $l=md+b, m\in {\mathbb{N}}^{*}, b\in \left[-\frac{d}{2}+1,\frac{d}{2}\right]\backslash \{0\}$. It is well known that the expected value ${\mu }_{{w}_{d}}$ of the linear combination ${w}_{d}$ of $d$ identically distributed random variables ${w}_{n}$:

$${w}_{d}(\theta )={a}_{0}{w}_{0}\left(\theta \right)+{a}_{1}{w}_{1}\left(\theta \right)+\dots +{a}_{d-1}{w}_{d-1}\left(\theta \right),$$

where ${a}_{n}$ are constants, is given by:

14 $${\mu }_{{w}_{d}}={\mathbb{E}}\left[{w}_{d}(\theta )\right]=\sum \limits_{n=0}^{d-1}{a}_{n}{\mu }_{{w}_{n}}={\mu }_{{w}_{n}}\sum \limits_{n=0}^{d-1}{a}_{n}.$$

Respectively, the variance ${\sigma }_{{w}_{d}}^{2}$ is given by:

15 $${\sigma }_{{w}_{d}}^{2}={\sigma }_{{w}_{n}}^{2}\sum \limits_{n=0}^{d-1}{a}_{n}^{2}.$$

Thus, the expected value ${\mu }_{{Y}_{l}}={\mathbb{E}}\left[{Y}_{l}\left(\theta \right)\right]$ of the DFT coefficients of Eq. (8) takes the form:

16 $${\mu }_{{Y}_{l}}={e}^{-j\frac{\pi l}{d}}\frac{1}{\pi l}{\mathbb{E}}\left[<{{\varvec{e}}}_{d,l},{{\varvec{v}}}_{d,l}\left(\theta \right)>\right]={e}^{-j\frac{\pi l}{d}}\frac{1}{\pi l}{\mu }_{{v}_{l}}\sum_{n=0}^{d-1}{e}_{n,l}, \quad l=md+b,$$

where ${\mu }_{{v}_{l}}={\mathbb{E}}\left[{v}_{n,l}\right]$. Moreover, for $l\ne md, \sum_{n=0}^{d-1}{e}_{n,l}=0$ and hence:

17 $${\mu }_{{Y}_{l}}=0.$$

Again, to determine whether ${\mu }_{{Y}_{l}}$ is a consistent estimator of the mean value of the DFT coefficient ${Y}_{l}(\theta )$, the variance ${\sigma }_{{Y}_{l}}^{2}$ needs to be calculated:

18 $${\sigma }_{{Y}_{l}}^{2}={\mathbb{E}}[\left({Y}_{l}\left(\theta \right)-{\mu }_{{Y}_{l}}{)}^{2}\right]={\mathbb{E}}\left[{\left|{e}^{-j\frac{\pi l}{d}}\frac{1}{\pi l}<{{\varvec{e}}}_{d,l},{{\varvec{v}}}_{d,l}\left(\theta \right)>\right|}^{2}\right]={\left(\frac{1}{\pi l}\right)}^{2}{\mathbb{E}}\left[{\left|<{{\varvec{e}}}_{d,l},{{\varvec{v}}}_{d,l}\left(\theta \right)>\right|}^{2}\right].$$

By using Eqs. (15) and (18) becomes:

19 $${\sigma }_{{Y}_{l}}^{2}={\left(\frac{1}{\pi l}\right)}^{2}{\sigma }_{{v}_{l}}^{2}\sum \limits_{n=0}^{d-1}{\left|{e}_{n,l}\right|}^{2}=\frac{1}{{\pi }^{2}{l}^{2}}d{\sigma }_{{v}_{l}}^{2}.$$

However, since $\frac{{\pi }^{2}{l}^{2}}{d}={\pi }^{2}\left({m}^{2}d+2mb+\frac{{b}^{2}}{d}\right)$ and:

20 $$\underset{d\to \infty }{{\text{lim}}}\left({m}^{2}d+2mb+\frac{{b}^{2}}{d}\right)\to \infty ,$$

we get:

21 $$\underset{d\to \infty }{{\text{lim}}}{\sigma }_{{Y}_{l}}^{2}\to 0,$$

from which it becomes evident that the variables ${Y}_{l}\left(\theta \right)$ constitute consistent estimators of the mean DFT values of the UPWM process.

Concluding, we have shown so far that for ergodic random input sequences of i.i.d. RV, the DFT coefficients on the side frequencies have zero expected values ${\mu }_{{Y}_{l}}=0$, while for the carrier harmonics the expected values are non-zero (Eq. 13). For this reason, the following analysis focuses on the DFT coefficients on the carrier harmonics only.

### Closed-form formulas for common distributions

As discussed in the “Introduction” section, if the GPDF (or equivalently the Probability Mass Function) of the RVs of the random input sequence ${{\varvec{x}}}_{d}\left(\theta \right)$ is known, then the DFT coefficients ${Y}_{md}\left(\theta \right)$ can also be expressed in closed form in terms of the moments of the RVs. To demonstrate this, distinct expressions are derived for the odd and even harmonics from Eq. (8):

22 $${Y}_{md}\left(\theta \right)=\left\{\begin{array}{l}\frac{{\left(-1\right)}^{\frac{m+1}{2}}}{\pi md}\sum_{n=0}^{d-1}{\text{cos}}\left(\pi m{x}_{n}\left(\theta \right)\right), \quad m \,odd\\ \frac{{\left(-1\right)}^\frac{m}{2}}{\pi md}\sum_{n=0}^{d-1}{\text{sin}}\left(\pi m{x}_{n}\left(\theta \right)\right), \quad m\, even \end{array}\right..$$

Using the Taylor series of the sinusoidal functions, we get:

23 $${Y}_{md}\left(\theta \right)=\left\{\begin{array}{l}\frac{{\left(-1\right)}^{\frac{m+1}{2}}}{\pi m}\sum_{k=0}^{\infty }\left(\frac{{\left(-1\right)}^{k}{\left(\pi m\right)}^{2k}}{\left(2k\right)!}\left(\frac{1}{d}\sum_{n=0}^{d-1}{x}_{n}^{2k}\left(\theta \right)\right)\right), \quad m {\text{ odd}}\\ \frac{{\left(-1\right)}^\frac{m}{2}}{\pi m}\sum_{k=0}^{\infty }\left(\frac{{\left(-1\right)}^{k}{\left(\pi m\right)}^{2k+1}}{\left(2k+1\right)!}\left(\frac{1}{d}\sum_{n=0}^{d-1}{x}_{n}^{2k+1}\left(\theta \right)\right)\right), \quad m \,even\end{array}\right..$$

Finally, assuming that $d$ is large enough, by the law of large numbers the sums:

$$\frac{1}{d}\sum_{n=0}^{d-1}{x}_{n}^{l}(\theta )={\mu }_{x,l}\left(\theta \right),$$

are consistent estimators of ${\mu }_{x,l}$ and hence, from Eq. (23) we get:

24 $${Y}_{md}={\mathbb{E}}\left[{Y}_{md}\left(\theta \right)\right]=\frac{1}{\pi m}\left\{\begin{array}{l}{\left(-1\right)}^{\frac{m+1}{2}}\sum_{k=0}^{\infty }\frac{{\left(-1\right)}^{k}}{\left(2k\right)!}{\left(\pi m\right)}^{2k}{\mu }_{x,2k}, \quad m\, odd\\ {\left(-1\right)}^\frac{m}{2}\sum_{k=0}^{\infty }\frac{{\left(-1\right)}^{k}}{\left(2k+1\right)!}{\left(\pi m\right)}^{2k+1}{\mu }_{x,2k+1}, \quad m \,even\end{array}\right..$$

**Figure 3:**
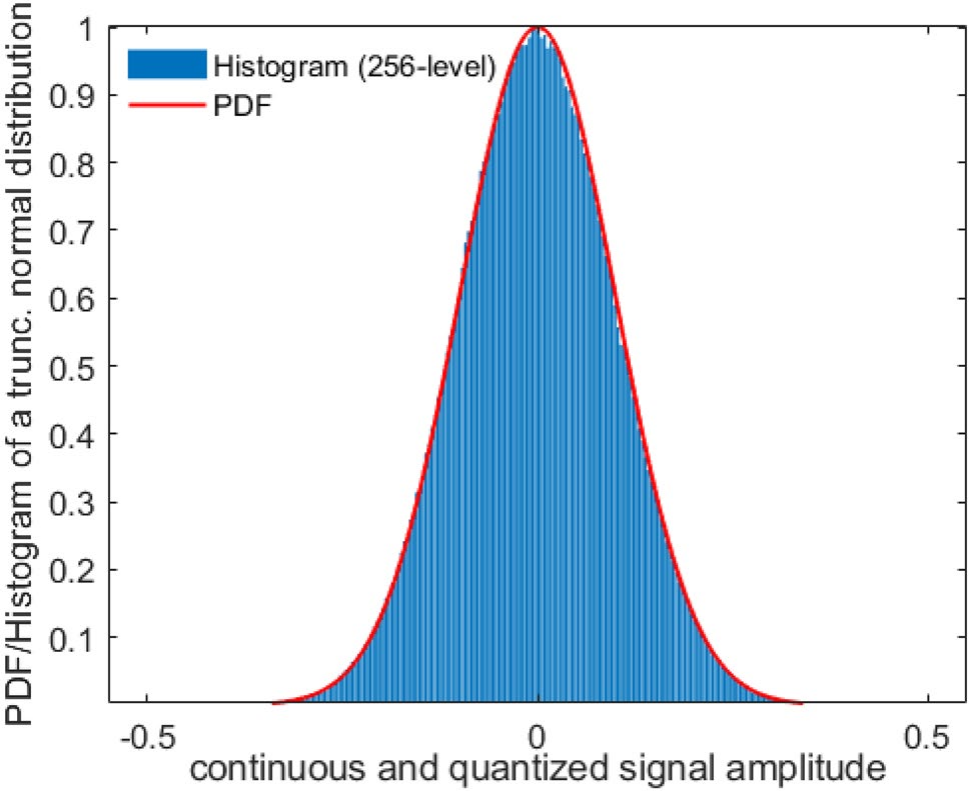
Example of approximating the truncated normal PDF via the respective 8-bit quantized distribution with 256 amplitude levels.

Equation (24) shows that the DFT coefficients of the UPWM signal on the carrier harmonics can be computed when the moments ${\mu }_{x,2k}$ of the random process are known. However, it is not particularly useful in this form since an infinite number of moments is required for its calculation, while at the same time, Eq. (24) only holds under the assumption of infinite order ergodicity, which is a hard constraint, thus making it unusual in practice. Nevertheless, there is a wide range of distributions parametrized by a small number of parameters, such as the truncated normal, truncated Laplacian, arcsine, beta and uniform to name a few, for which Eq. (24) becomes practically useful. Significantly, as we are going to demonstrate in the next subsections, for such input signal distributions Eq. (24) leads to closed-form formulas that allow for computation of the DFT coefficients of the underlying UPWM random process. However, their use demands knowledge of the parameters’ values, which is often not available in real world applications. This obstacle can be overcome by estimating these parameters. In this case, a closed-form estimator formula of the DFT coefficients of the UPWM signal is derived from Eq. (24) just by replacing the unknown parameters values by their estimations from the input signal. This can be practically done since the needed RV moments before quantization can be estimated from their quantized counterparts, provided that the quantization resolution is sufficiently high and the vast majority of the RV values lies within the desired interval^38^. Indeed, the statistics of the RVs, along with the 256 levels used for quantization of the values in the interval [− 1/2, 1/2], ensure that the aforementioned constraints hold and thus, the resulting GPDF (or equivalently the Probability Mass Function (PMF)) closely approximates the continuous one. An example demonstrating this fact for the truncated normal distribution $N(0, 0.01)$ is shown in Fig. 3. In “Experimental evaluation” section, these aspects are validated by the excellent agreement between the computational evaluations and the analytical estimations via the proposed estimator-form formulas.

#### Symmetric distributions

Let us now concentrate on the derivation of closed-form expressions of the spectral lines ${Y}_{md}$ for typical symmetric distributions, and specifically for the truncated normal and Laplacian distributions, the uniform distribution and the arcsine distribution. Then, their estimator-form counterparts are obtained by replacing the unknown parameters’ values with their estimations from the quantized input signals.

To this end, let us begin from the truncated normal distribution $N(0, {\sigma }^{2})$ whose even moments ${}_{N}{\mu }_{x,2k}$ can be expressed with respect to the variance ${}_{\text N}{\sigma }_{x}^{2}$ as:

$${}_{N}{\mu }_{x,2k}={ }_{\text N}{\sigma }_{x}^{2k}\frac{\left(2k\right)!}{k!{2}^{k}}.$$

By substituting in Eq. (24), we get:

$${}_{N}{Y}_{md}=\frac{1}{\pi m}{\left(-1\right)}^{\frac{m+1}{2}}\left(1+\sum_{k=1}^{\infty }{\left(-1\right)}^{k} {\left(m\pi \right)}^{2k}\frac{{ }_{\text N}{\sigma }_{x}^{2k}}{\left(2k\right)!!}\right), \quad m\, \text{ odd},$$

which, after some simple manipulations, leads to:

25 $${}_{N}{Y}_{md}=\frac{{\left(-1\right)}^{\frac{m+1}{2}}}{\pi m}{e}^{-\frac{{\pi }^{2}{ m}^{2}{ }_{\text N}{\sigma }_{x}^{2}}{2}}, \quad m\,\text{ odd}.$$

In practice, Eq. (25) cannot be used for the computation of ${}_{N}{Y}_{md}$ except if the variance ${}_{\text N}{\sigma }_{x}^{2}$ is known. If ${}_{\text N}{\sigma }_{x}^{2}$ is unknown, it can be estimated from the input signal ${x}_{n}(\theta )$. By replacing it in Eq. (26), the estimator-form counterpart is obtained:

26 $${}_{N}{Y}_{md}\left(\theta \right)=\frac{{\left(-1\right)}^{\frac{m+1}{2}}}{\pi m}{e}^{-\frac{{\pi }^{2}{ m}^{2}{ }_{\text N}{\sigma }_{x}^{2}\left(\theta \right)}{2}}, \quad m\,\text{ odd},$$

which we are going to use for the estimation of the coefficients ${}_{N}{Y}_{md}$.

Following similar steps and by taking into account that the moments for the truncated Laplacian, the symmetric uniform and the arcsine distribution with values in the interval $\left[- \,0.5, 0.5\right]$, corresponding to scale parameter ${}_{S}{w}_{x}=1,$ are given by the following relations:

- ${ }_{L}{\mu }_{x,2k}={ }_{L}{b}_{x}^{2}\left(2k\right)!$, where${ }_{L}{b}_{x}$ is the Laplace parameter,
- ${\mu }_{x,2k}=\frac{{ }_{U}{a}_{x}^{2k}}{\left(2k+1\right)}$, where${ }_{U}{a}_{x}$ is the upper limit of the distribution, and
- $${ }_{S}{\mu }_{x,2k}=\frac{\left(2k-1\right)!!}{\left(2k\right)!!}$$.

The closed-form expressions for these distributions become:

27 $${}_{L}{Y}_{md} = \frac{1}{\pi m}{\left(-1\right)}^{\frac{m+1}{2}}\frac{1}{1+{\left(m\pi { }_{L}{b}_{x}\right)}^{2}}, \quad m\text{ odd},$$

28 $${}_{N}{Y}_{md}=\frac{1}{\pi m}{\left(-1\right)}^{\frac{m+1}{2}}\frac{{\text{sin}}\left(m\pi { }_{U}{a}_{x}\right)}{m\pi { }_{U}{a}_{x}}, \quad m\text{ odd},$$

29 $${}_{S}{Y}_{md}=\frac{1}{\pi m}{\left(-1\right)}^{\frac{m+1}{2}}{J}_{0}\left(m\pi \right), \quad m\text{ odd},$$

with ${J}_{0}\left(.\right)$ denoting the Bessel function of the first kind.

For the two first distributions, their estimator-form counterparts are given by:

30 $${}_{L}{Y}_{md}\left(\theta \right)= \frac{1}{\pi m}{\left(-1\right)}^{\frac{m+1}{2}}\frac{1}{1+{\left(m\pi { }_{L}{b}_{x}\left(\theta \right)\right)}^{2}}, \quad m\text{ odd},$$

31 $${}_{N}{Y}_{md}=\frac{1}{\pi m}{\left(-1\right)}^{\frac{m+1}{2}}\frac{{\text{sin}}\left(m\pi { }_{U}{a}_{x}(\theta )\right)}{m\pi { }_{U}{a}_{x}(\theta )}, \quad m\text{ odd},$$

respectively. From Eq. (29) it becomes evident that the resulting closed-form expression for the DFT coefficients for the arcsine distribution do not depend on any statistic of the input signal. Thus, Eq. (29) can be directly used for the computation of the coefficients. It should be noted that Eq. (29) agrees with the relation obtained by the double Fourier series method proposed in Ref.^24^. Finally, it is important to highlight that since the computation of ${}_{\text N}{\sigma }_{x}^{2}(\theta )$, ${}_{L}{b}_{x}(\theta )$ and ${}_{U}{a}_{x}\left(\theta \right)$ has a complexity $O\left(d\right)$, the use of the above estimator-forms allows for the estimation of the coefficients ${Y}_{md}$ in linear time, as opposed to the $O\left({D}^{2}\right)$ and $O\left(D{\text{log}}\left(D\right)\right)$ complexity of the DFT and FFT algorithms, respectively.

#### Non-symmetric distributions

The potential of the presented analysis for the estimation of the DFT of UPWM signals derived from stochastic input sequences from closed-form formulas, is not restricted to inputs following single-parameter (neglecting the mean) symmetric distributions but it actually extends to non-symmetric ones. This is demonstrated in this subsection by deriving such a closed-form formula for the two-parameter non-symmetric beta distribution. The probability density function of the beta distribution is given by:

$$f\left(x\right)=\frac{1}{B\left(a,b\right)}{x}^{a-1}{\left(1-x\right)}^{b-1}, x\in \left(\text{0,1}\right),$$

where $B\left(\cdot ,\cdot \right)$ the beta function and $a, b$ the “left” and “right” distribution parameters. The moments ${}_{B}{\mu }_{x,k}$ of the beta distribution are expressed in terms of the two parameters as:

$${}_{B}{\mu }_{x,k}=\frac{B\left(a+k,b\right)}{B\left(a,b\right)}.$$

Since the distribution takes values in the range $x\in (\text{0,1})$, Eq. (8) of the UPWM DFT must be adapted as follows:

32 $${{\text{Y}}}_{{\text{md}}}\left(\uptheta \right)=\frac{{{\text{A}}}_{{\text{m}}}}{{\text{d}}}\sum_{{\text{n}}=0}^{{\text{d}}-1}\mathit{sin}\left(\pi \text{m}{{\text{x}}}_{{\text{n}}}\left(\uptheta \right)\right).$$

Following the same procedure as in “Closed-form formulas for common distributions” section, we get the following counterpart of Eq. (24):

33 $${{\text{Y}}}_{{\text{md}}}=\frac{{\left(-1\right)}^{{\text{m}}}}{\pi \text{m}}\sum_{{\text{k}}=0}^{\infty }\frac{{\left(-1\right)}^{{\text{k}}}}{\left(2{\text{k}}+1\right)!}{\left(\pi \text{m}\right)}^{2{\text{k}}+1}{ }_{{\text{B}}}{\upmu }_{{\text{x}},2{\text{k}}+1}.$$

Note that in this case, $m$ can be either even or odd. Now, substituting through ${}_{B}{\mu }_{x,k}$ in Eq. (34) we get:

$${{\text{Y}}}_{{\text{md}}}=\frac{{\left(-1\right)}^{{\text{m}}}}{\pi \text{m}}\sum_{{\text{k}}=0}^{\infty }\frac{{\left(-1\right)}^{{\text{k}}}}{\left(2{\text{k}}+1\right)!}{\left(\pi \text{m}\right)}^{2{\text{k}}+1}\frac{{\text{B}}\left({\text{a}}+2{\text{k}}+1,{\text{b}}\right)}{{\text{B}}\left({\text{a}},{\text{b}}\right)},$$

which leads to the closed-form expression:

34 $${{\text{Y}}}_{{\text{md}}}=\frac{{\text{B}}\left({\text{a}}+1,{\text{b}}\right)}{\text{B}\left({\text{a}},{\text{b}}\right)}{ }_{2}{{\text{F}}}_{3}\left(\frac{{\text{a}}+1}{2},\frac{{\text{a}}}{2}+1;\frac{3}{2},\frac{{\text{a}}+{\text{b}}+1}{2}, \frac{{\text{a}}+{\text{b}}}{2}+1;-\frac{{\left(\text{m}\pi \right)}^{2}}{4}\right),$$

where ${}_{2}{F}_{3}(\cdot )$ the generalized hypergeometric function.

Actually, Eq. (34) cannot be used for the desired computations since the values of the parameters *α* and *b* are unknown. In order to obtain the estimator-form counterpart of Eq. (34) we can estimate the values of the aforementioned parameters from the input signal ${x}_{n}\left(\theta \right)$ by using the moments method^39^, that is:

$${\alpha }\left(\uptheta \right)={\upmu }_{{\text{x}}}\left(\uptheta \right)\left(\frac{{\upmu }_{{\text{x}}}\left(\uptheta \right)\left(1-{\upmu }_{{\text{x}}}\left(\uptheta \right)\right)}{{\upsigma }_{{\text{x}}}^{2}\left(\uptheta \right)}-1\right),$$

$${\text{b}}(\uptheta )={(1-\upmu }_{{\text{x}}}(\uptheta ))\left(\frac{{\upmu }_{{\text{x}}}\left(\uptheta \right)\left(1-{\upmu }_{{\text{x}}}\left(\uptheta \right)\right)}{{\upsigma }_{{\text{x}}}^{2}\left(\uptheta \right)}-1\right),$$

and substitute them into Eq. (34) to obtain its estimator-form counterpart:

35 $${{\text{Y}}}_{{\text{md}}}\left(\uptheta \right)=\frac{{\text{B}}\left({\text{a}}\left(\uptheta \right)+1,{\text{b}}\left(\uptheta \right)\right)}{\text{B}\left({\text{a}}\left(\uptheta \right),{\text{b}}\left(\uptheta \right)\right)}{ }_{2}{{\text{F}}}_{3}\left(\frac{{\text{a}}(\uptheta )+1}{2},\frac{{\text{a}}(\uptheta )}{2}+1;\frac{3}{2},\frac{{\text{a}}(\uptheta )+{\text{b}}(\uptheta )+1}{2}, \frac{{\text{a}}(\uptheta )+{\text{b}}(\uptheta )}{2}+1;-\frac{{\left(\text{m}\pi \right)}^{2}}{4}\right).$$

Up to this point, all quantities and relations regarding the DFT analysis of UPWM signals generated by stochastic inputs have been defined and derived. In the next section, experimental results are presented, demonstrating the validity of the proposed model.

## Experimental evaluation

In this section, the validity of the presented model is established through the demonstration of its predictive accuracy concerning:

- The effect of the observation window size on the side frequency components of the UPWM DFT.
- The accuracy of the closed-form formulas and their estimator-forms presented in “Closed-form formulas for common distributions” section, evaluated against the direct computational evaluations of the DFT.
- The effect of the distribution’s asymmetry on the even carrier harmonics.
- The application of the estimator-form formula of the truncated Laplacian distribution in the estimation of the DFT of a UPWM signal originating from a real speech signal.

All PCM signals used for the evaluations are sampled at 44.1 kHz and quantized with 8-bit resolution.

### The effect of the observation window size

In “Random input sequences” section it was shown that the side frequency DFT coefficients ${Y}_{l}\left(\theta \right)$ of a UPWM signal generated from an input sequence ${{\varvec{x}}}_{d}\left(\theta \right)$ with i.i.d. RV ${x}_{n}\left(\theta \right)$ have zero mean (Eq. 17) and a variance given by Eq. (19). This aspect is proved and demonstrated here by computational evaluation of the DFT of the sequence ${y}_{{n}^{\prime}}$ generated from random input signals ${{\varvec{x}}}_{d}\left(\theta \right)$ with different distributions, particularly truncated normal, truncated Laplacian and symmetric uniform with parameters ${}_{\text N}{\sigma }_{x}=0.1, { }_{L}{b}_{x}=0.05, { }_{U}{a}_{x}=\frac{1}{2}$ respectively, and the arcsine distribution.

To this end we are going to use two windows of size ${d}_{1} {\text{and}} {d}_{2} {\text{respectively}}$. Then, according to Eq. (19), the following relation holds:

$$\frac{{\sigma }_{{Y}_{m{d}_{2}+{b}_{2}}}^{2}}{{\upsigma }_{{{Y}_{m{d}_{1}+b}}_{1}}^{2}}=\frac{{l}_{1}^{2}}{{l}_{2}^{2}}\frac{{d}_{2}{\sigma }_{{v}_{{l}_{2}}}^{2}}{{d}_{1}{\sigma }_{{v}_{{l}_{1}}}^{2}}\cong \frac{{d}_{1}{\sigma }_{{v}_{{l}_{2}}}^{2}}{{d}_{2}{\sigma }_{{v}_{{l}_{1}}}^{2}},$$

where ${l}_{1}=m{d}_{1}+{b}_{1}$ and ${l}_{2}=m{d}_{2}+{b}_{2}$ respectively, and ${\sigma }_{{Y}_{md+b}}^{2}={\mathbb{E}}\left[{\left|{Y}_{md+b}\left(\theta \right)\right|}^{2}\right]$.

In order to evaluate the ratio $\frac{{\sigma }_{{v}_{{l}_{2}}}^{2}}{{\sigma }_{{v}_{{l}_{1}}}^{2}}$, we assume a side frequency $f$ and the fact that RVs ${v}_{{l}_{i}}$ are given by:

36 $${v}_{{l}_{i}}={\text{sin}}\left(\left(\pi m+\frac{{b}_{i}}{{d}_{i}}\right)\left({x}_{n}+\frac{1}{2}\right)\right), \quad i=1, 2.$$

However, it is easy to see that for the same frequency $f$, the ratios $\frac{{b}_{i}}{{d}_{i}}, i=\text{1,2}$ are equal, since:

$$\frac{f}{{f}_{s}}=\frac{{l}_{1}}{{d}_{1}}=\frac{{l}_{2}}{{d}_{2}},$$

and thus,

$$\frac{{b}_{1}}{{d}_{1}}=\frac{{b}_{2}}{{d}_{2}}.$$

Hence, ${\sigma }_{{v}_{{l}_{1}}}^{2}={\sigma }_{{v}_{{l}_{2}}}^{2}$ and from Eq. (34) we get:

$$\frac{{\sigma }_{{Y}_{m{d}_{2}+{b}_{2}}}^{2}}{{\upsigma }_{{{Y}_{m{d}_{1}+b}}_{1}}^{2}}\cong \frac{{d}_{1}}{{d}_{2}}.$$

To computationally evaluate this reduction, let us consider the vector ${{\varvec{Y}}}_{md+b}\left(\theta \right)=\left[{Y}_{md-\frac{d}{2}+1}\left(\theta \right), {Y}_{md-\frac{d}{2}+1}\left(\theta \right),\dots ,{Y}_{md+\frac{d}{2}}\left(\theta \right)\right]$ that contains $d-1$ RVs ${Y}_{l}(\theta )$ and use it for the estimation of the variance ${\sigma }_{{Y}_{md+b}}^{2}$ via its weighted squared ${l}_{2}$ norm, i.e.:

37 $${\tilde{\sigma }}_{{Y}_{md+b}}^{2}\left(\theta \right)=\frac{1}{d-1}{||{{\varvec{Y}}}_{md+b}\left(\theta \right)||}_{2}^{2}.$$

**Figure 4:**
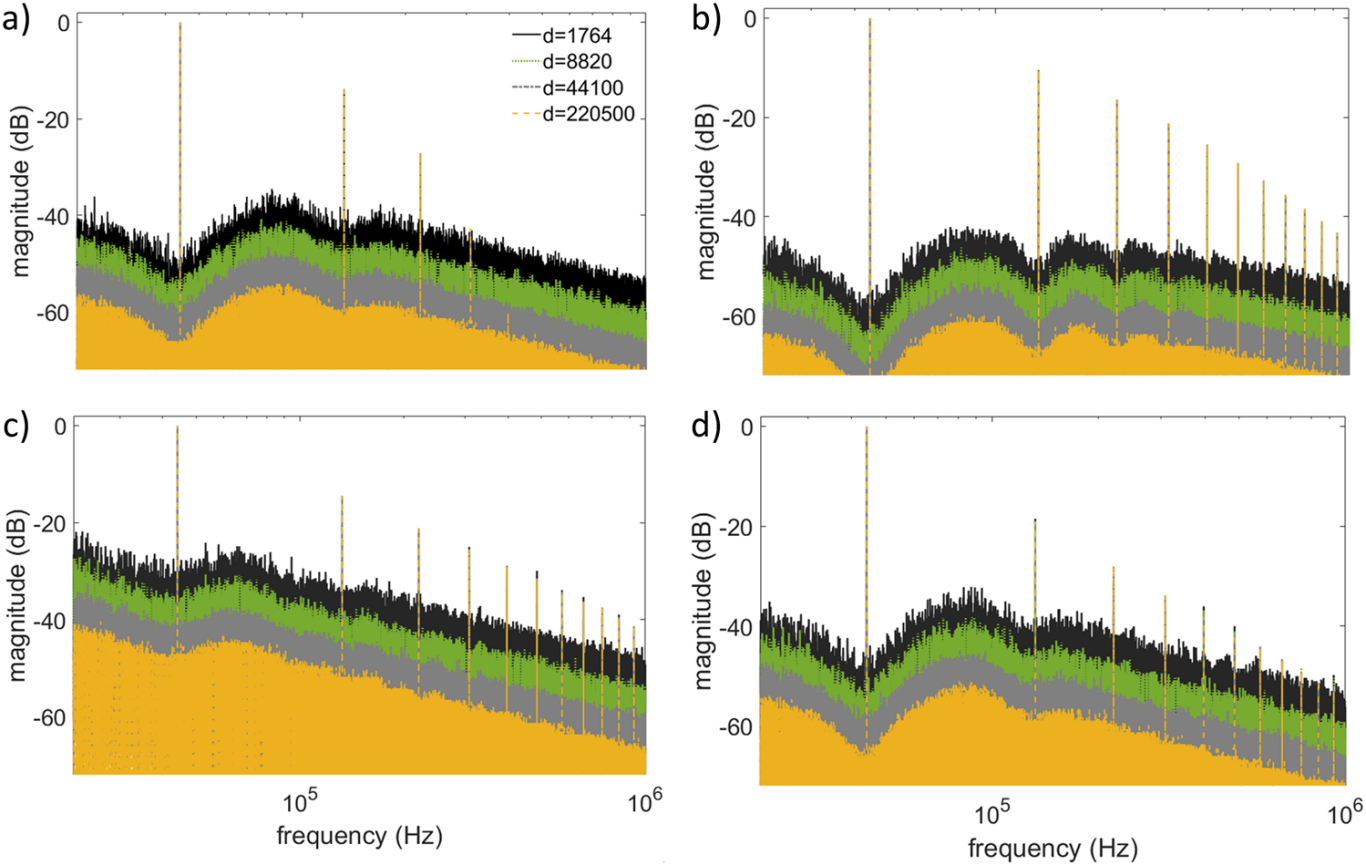
DFT spectra of UPWM signals generated from input signals with (**a**) truncated normal, (**b**) truncated Laplacian, (**c**) arcsine and (**d**) uniform distributions, evaluated for four (4) observation windows, ${d}_{1}=\frac{\text{44,100}}{{5}^{2}}=1764, {d}_{2}=\frac{\text{44,100}}{5}=8820, {d}_{3}=\text{44,100}, {d}_{4}=220,500=44,100.5$.

The obtained results are presented in Fig. 4, where the out-of-band DFT spectra ${Y}_{l}\left(\theta \right)$ of the UPWM signals are shown for four (4) observation windows of duration ${d}_{1}=\frac{\text{44,100}}{{5}^{2}}=1764, {d}_{2}=\frac{\text{44,100}}{5}=8820, {d}_{3}=\text{44,100}, {d}_{4}=\text{220,500}=\text{44,100} \times 5$ samples, corresponding to 0.04, 0.2, 1 and 5 s, respectively. The total energy of the signals ${E}_{{\text{tot}}}=||{{\varvec{x}}}_{d}\left(\theta \right)|{|}_{2 }^{2}$ is kept constant for the results to be comparable. It is evident that the variance in the coefficients ${Y}_{l}$ on the side frequencies is noticeably reduced with increasing window size.

In general, the average variance on the side frequencies of any carrier harmonic $m$ between two consecutive windows with the ratio of their lengths equal to $a$ is reduced by a factor of $a$, or equivalently $20{{\text{log}}}_{10}\left(a\right){\text{dB}}$. The average variance on the side frequencies around the 1st UPWM carrier harmonic, estimated via Eq. (37) and calculated via Eq. (19), for the four input signals computed for four window sizes with $a=5$, are given in Table 1.

**Table 1:** Estimated (${\tilde{\sigma }}_{{Y}_{md+b}}^{2}$(*θ*)) and calculated (${\sigma }_{{Y}_{{Y}_{md+b}}}^{2}$) variance of the side frequencies around the 1st UPWM carrier harmonic for four input signals with four different window sizes.

Signal/number of samples	${\tilde{\sigma }}_{{Y}_{md+b}}^{2}$(*θ*) vs ${\tilde{\sigma }}_{{Y}_{md+b}}^{2}$							
	1764		8820		44,100		220,500	
	Equation (37)	Equation (19)	Equation (37)	Equation (19)	Equation (37)	Equation (19)	Equation (37)	Equation (19)
Arcsine	3.74E−05	3.81E−05	7.78E−06	7.64E−06	1.49E−06	1.50E−06	2.99E−07	3.00E−07
Normal	5.00E−06	5.02E−06	9.84E−07	1.01E−06	2.03E−07	2.00E−07	4.10E−08	4.01E−08
Laplace	2.36E−06	2.60E−06	5.43E−07	4.97E−07	1.05E−07	1.03E−07	2.10E−08	2.06E−08
Uniform	2.82E−05	2.90E−05	5.77E−06	5.77E−06	1.17E−06	1.15E−06	2.40E−07	2.30E−07

### Non-symmetric distributions

Here it is shown that asymmetry in the GPDF leads to the formation of UPWM DFT components on the even harmonics, as predicted by Eq. (23). To demonstrate this aspect, computational evaluations are presented for 5 different input signals generated from the four-parameter beta distribution with different asymmetry levels. For the distributions, the following parameters were selected:

$$\mu =0, \sigma =1,{\mu }_{x,4}=3,$$

while the skewness ${\mu }_{x,3}$ took the values:

$${\mu }_{x,3}=0.2, 0.4, 0.6, 0.8, 1.$$

**Figure 5:**
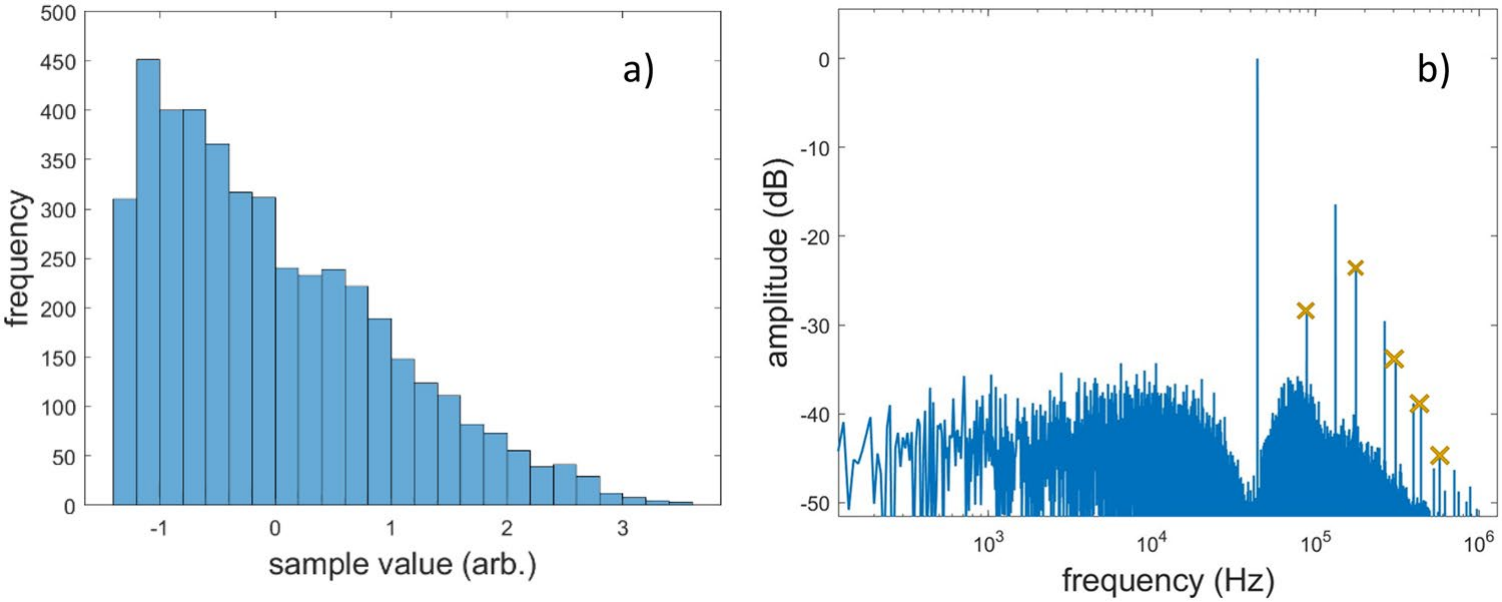
(**a**) histogram of a signal ${x}_{d}$ with samples following a Pearson distribution with parameters $\mu =0, \sigma =1, {\mu }_{x,3}=0.8, {\mu }_{x,4}=3$ and (**b**) DFT spectra of the UPWM signal generated from ${{\varvec{x}}}_{d}$, where the even harmonics are denoted with cross markers.

**Figure 6:**
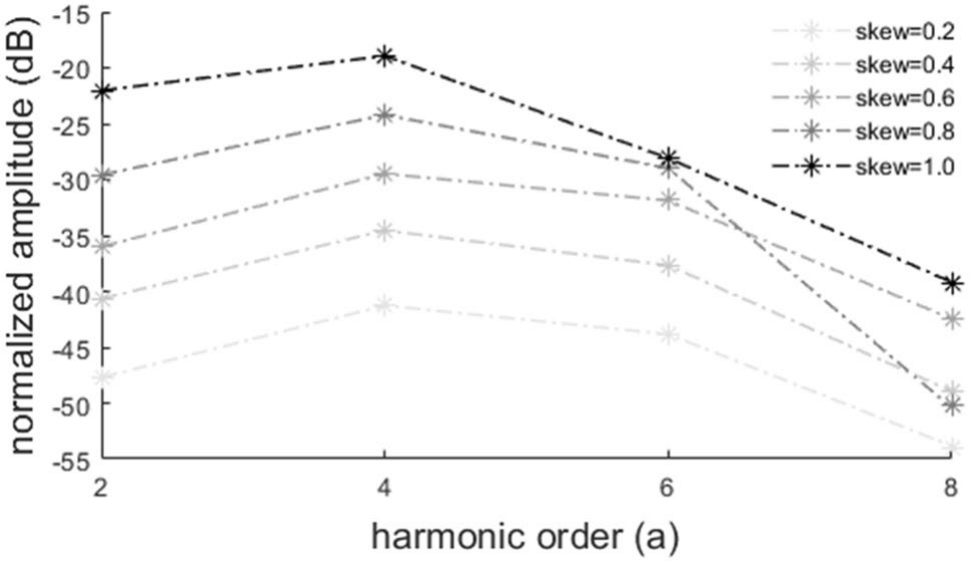
DFT coefficients (in dB) of the even carrier harmonics of five UPWM signals generated by an equal number of random input signals whose samples follow Pearson distributions with parameters ${\mu }_{x}=0, {\sigma }_{x}=1, {\mu }_{x,4}=3$ and ${\mu }_{x,3}=0.2, 0.4, 0.6, 0.8, 1$.

For the signals, an observation window of 44,100 samples was used, corresponding to 1sec duration. Figure 5a shows the histogram of an input signal generated for ${\mu }_{x,3}=0.8$ while Fig. 5b shows the DFT spectrum of the corresponding UPWM signal. It becomes evident that the skewed distribution exhibits prominent DFT coefficients on the even carrier harmonics which are completely absent in the symmetric distributions (see “Non-symmetric distributions” section). Moreover, Fig. 6 shows the magnitude (in dB) of the DFT coefficients on the 4 first even harmonics for the 5 different UPWM signals, normalized with respect to the coefficient of the carrier fundamental. Generally, the coefficients are amplified with increasing skewness, with the only exception being the coefficient of the 8th harmonic for ${\mu }_{x,3}=0.8$, which could be due statistical error. Although the results for the particular distribution show an increase in the even harmonics with increasing skewness, no generalization can be made for other skewed distributions.

### Closed-form formulas

In this subsection we demonstrate the validity of the closed-form formulas for the estimation of the DFT coefficients presented in “Closed-form formulas for common distributions” section by comparing them with direct computational evaluations via DFT. For this purpose, the truncated normal, truncated Laplacian, uniform and arcsine distributions are used with the parameter values given in “The effect of the observation window size” section, as well as the two-parameter beta distribution presented in “Non-symmetric distributions” section with $a=1$ and $b=3$. All the simulated signals have $d=\text{44,100}$ samples. The results of the comparative evaluations are summarized in Table 2 where “DFT” denotes the computational evaluations via DFT and “CFEst” and “CFF” denote the estimations and calculations obtained via the estimator-form and the closed-form formulas of “Closed-form formulas for common distributions” section, respectively. The values in Table 2 correspond to magnitude in dB of the DFT coefficients on the first 4 odd carrier harmonics (3, 5, 7 and 9) for the symmetric distributions and the first 4 harmonics (2, 3, 4, 5) for the beta distribution, normalized for each signal with respect to the coefficient of the fundamental frequency. From Table 2 it becomes evident that the estimations via the closed-form formulas and the estimator forms are in excellent agreement with the directly evaluated DFT coefficients, with deviations of less than 1.5 dB. This proves that, when the input signal’s samples are i.i.d. RVs with known distribution, the DFT spectra of the UPWM signal observed within a sufficiently large window can be estimated from the distribution’s statistical parameters, given an ergodic generating process.

**Table 2:** Comparison of DFT coefficients directly computed via DFT (DFT), estimated by the estimator-form formulas (CFEst) and calculated via the closed-form formulas (CFF).

Signal	Harmonic order (α)											
	3			5			7			9		
	DFT	CFEst	CFF	DFT	CFEst	CFF	DFT	CFEst	CFF	DFT	CFEst	CFF
Arcsine	− 14.54	–	− 14.04	− 21.3	–	− 20.65	− 25.73	–	− 25.01	− 29.20	–	− 28.27
Normal	− 13.02	− 13.01	− 12.97	− 24.46	− 24.39	− 24.27	− 37.95	− 37.73	− 37.48	− 53.83	− 53.79	− 53.38
Laplace	− 11.05	− 11.05	− 11.05	− 17.87	− 17.89	− 17.88	− 23.47	− 23.50	− 23.48	− 28.28	− 28.32	− 28.30
Uniform	− 19.19	− 19.09	− 19.09	− 28.14	− 27.96	− 27.96	− 33.87	− 33.80	− 33.80	− 38.39	− 38.17	− 38.17

	2			3			4			5		
Beta	− 7.50	− 7.58	− 7.53	− 14.85	− 15.03	14.98	− 19.52	− 19.57	− 19.60	− 23.56	− 23.63	23.59

It is important to repeat here that, while computational complexity of the typical DFT and FFT algorithms is $O({D}^{2})$ and $O(D{\text{log}}(D))$, respectively, estimation of the UPWM spectra on the carrier harmonics via Eq. (12) can be done in linear time $O(d)$, where $d, D={M}_{p}d$ are the number of samples of the input and PWM signals, respectively. Of course, estimation via the closed-form formulas does not depend on $d$, considering that the statistical parameters of the underlying process are known. Otherwise, linear time is required for their estimation from a realization of the process.

### Application on a real speech signal

The truncated Laplacian distribution is commonly used to describe sparse signals e.g., speech signals among others^33–35^. Consequent samples of speech signal are correlated and hence cannot be considered as i.i.d. RVs. Thus, Eq. (19) does not hold and the DFT components ${Y}_{l}(\theta )$ of the side frequencies cannot be estimated from the statistics of the input signal. However, even in such a case, the carrier harmonics and particularly the odd harmonics which dominate the spectral energy of the out-of-band range, can be predicted by the presented model with sufficient accuracy. From Eq. (11) it can be seen that the ${Y}_{md}(\theta )$ do not depend on the particular sequence of ${x}_{n}(\theta )$ but rather on their sum, so that any rearrangement of the variables in a non-stochastic manner that introduces dependency between the variables does not affect the value of the estimator.

**Figure 7:**
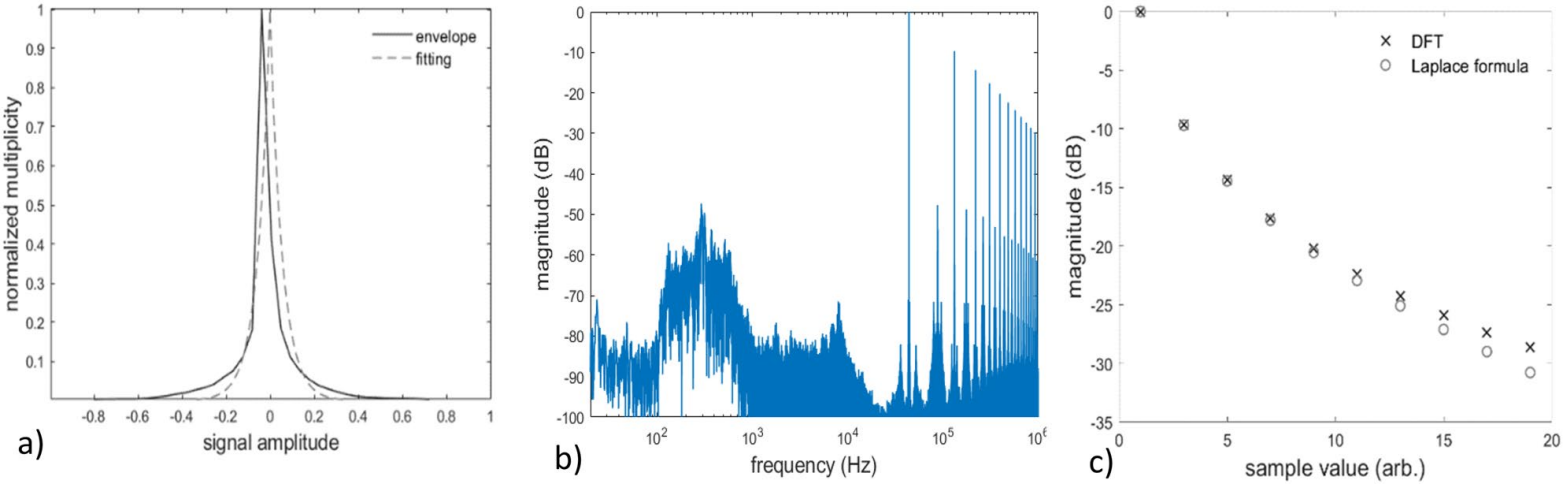
(**a**) envelope and Laplace fitting curve of the histogram and (**b**) computationally evaluated DFT spectrum of a 5s female speech extract; (**c**) comparative estimation of the DFT coefficients via DFT of the UPWM signal and via the closed-form Laplace formula.

To demonstrate this aspect, a female speech extract with 5s duration is used, taken from the Archimedes anechoic audio library^40^. The histogram of the extract is calculated and fitted via a Laplace function, to determine the distribution’s width parameter. Then, the coefficients ${Y}_{md}(\theta )$ are estimated via Eq. (27) and the results are compared with the direct calculation of the UPWM signal’s DFT. Figure 7a shows the histogram’s envelope and the fitting curve of the first extract, while Fig. 7b shows the DFT spectrum of the respective UPWM signal. In Fig. 7b, the prominence of the odd carrier harmonics DFT components becomes evident. Even carrier harmonics are also present, however, they have significantly less energy compared to the odd harmonics. Finally, Fig. 7c presents the comparative results of the DFT coefficients via DFT of the UPWM signal and via the closed-form formula of Eq. (27). It can be seen that the coefficients ${Y}_{md}(\theta )$ of the odd carrier harmonics are predicted by ${}_{L}{Y}_{md}$ with high precision, demonstrating the applicability of the proposed analysis in real PWM systems.

## Conclusions

This work proposed a mathematical framework for the analysis and design of PWM systems whose input signals can be modeled as i.i.d random sequences, by demonstrating that the resulting UPWM DFT spectrum in the out-of-band frequency range can be precisely estimated from the statistics of the input sequence. It also demonstrated that this approach is applicable in real-world PWM systems with input signals such as speech, music or other communication signals. Particularly, first it was proven that the expected values of the DFT coefficients are consistent estimators of the true mean, with a zero value for all out-of-band frequencies except for the carrier harmonics. For random input signals whose moments can be expressed in terms of a few parameters of the distribution, such as the truncated normal, truncated Laplacian, uniform, arcsine and the asymmetric beta distribution, closed-form formulas were derived for the estimation of the carrier harmonics DFT coefficients. The results were validated by comparison to computational evaluations of the DFT of PWM signal, showing very good agreement.

Hence, the proposed method allows for precise estimation of the excessive out-of-band energy of UPWM signals generated from input sequencies of i.i.d. RV, solely based on the statistical characteristics of the RV. It is worth noting that in the presented case, estimation of the DFT components can be generally done in linear time $O(d)$ as opposed to typical $O({D}^{2})$ of the DFT and $O(D\cdot {\text{log}}(D))$ of the FFT. The theoretical interest of such a finding is complemented by significant practical interest due to the applicability of stochastic modeling of signals that constitute common inputs to PWM systems, e.g., speech, music, sonar and noise signals among others. The capability to derive closed-form formulas, indicatively demonstrated for the abovementioned distributions, can significantly reduce computational complexity and load. The postulated analysis can significantly facilitate hardware design and signal processing optimization of PWM systems, especially for the mitigation of the hazards and restrictions owed to the excessive out-of-band energy of the UPWM signals. Finally, input-output statistical correlation in other non-linear signal transformations will be investigated in the future.

## Data Availability

The data analysed during the current study are available from the corresponding author on reasonable request.
